# Response to the letter to the editor entitled “Recurrent Acute Pancreatitis by Food Allergy?”

**DOI:** 10.5415/apallergy.0000000000000167

**Published:** 2025-01-08

**Authors:** Hideo Kaneko

**Affiliations:** 1 Department of Pediatric Medical Care, Gifu Prefectural General Medical Center, Gifu, Japan; 2 Department of Medical Genetics, Gifu Prefectural General Medical Center, Gifu, Japan

## To the Editor,

I thank Dr. Öner Özdemir for his valuable comments on our case of recurrent acute pancreatitis in a patient with a peanut allergy [1]. I have addressed his concerns in the following response.

**Figure 1.**
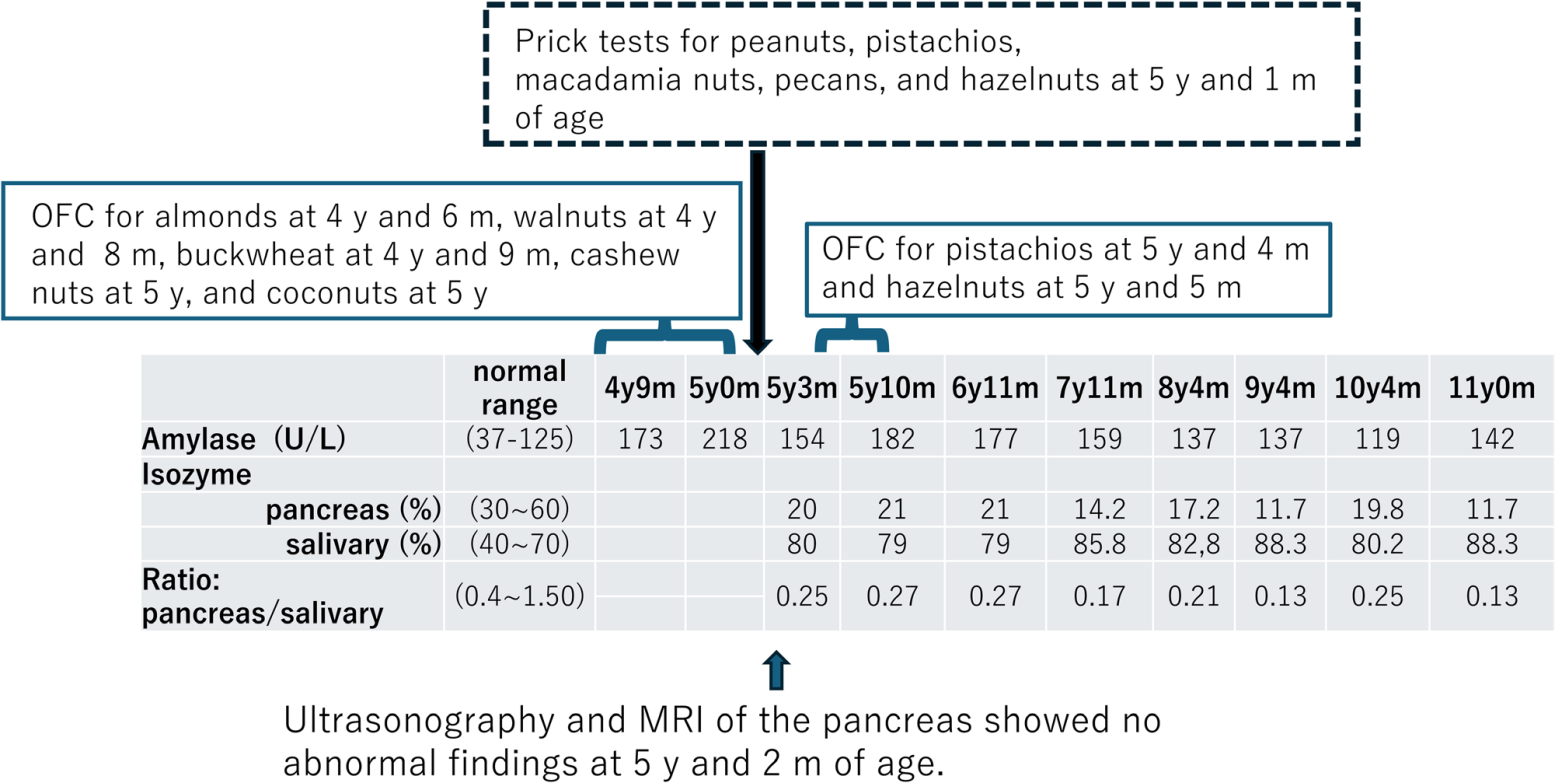
Long-term course of serum amylase and isoenzymes, skin prick tests, and oral food challenge tests (OFC). The skin prick tests for nuts and buckwheat, conducted prior to the OFC, were negative. Saline served as the negative control for the skin prick test and resulted in only needle marks. All OFCs for nuts and buckwheat, except for peanuts, yielded negative results. m, months; y, years.

Regarding our hospital’s standards, the normal ranges for serum amylase and pancreatic amylase values are 37 to 125 IU/L and 16 to 52 IU/L, respectively. The patient’s baseline serum amylase level was slightly elevated; therefore, we repeatedly assessed serum amylase and amylase isozyme levels (Fig. 1). The results indicated that the slightly elevated serum amylase levels could be attributed to the salivary gland, not the pancreas. Based on these results, benign pancreatic hyperenzymemia and macroamylasemia were ruled out [2]. At 5 years and 2 months of age, magnetic resonance imaging (MRI) and ultrasonography of the pancreas did not reveal any signs of chronic pancreatitis, despite MRI being highly sensitive and specific for this diagnosis [3]. Computed tomography of the salivary glands, performed by an otolaryngologist, showed no abnormalities.

The etiology of salivary amylasemia in this patient remains unclear. Blood glucose and serum HbA1c levels were within normal ranges. The patient was able to attend elementary and junior high school without any intellectual development issues. Additionally, the patient did not exhibit digestive symptoms such as abdominal pain or diarrhea. Although pancreatic exocrine and endocrine functions were not examined, we assumed that they were normal owing to the absence of suggestive symptoms of dysfunction. These findings indicate that the patient did not have chronic pancreatitis and that the pancreas was normal, except when peanuts were ingested.

Specific IgE levels for nuts were low in this patient. The skin prick tests for nuts and buckwheat, conducted prior to the oral food challenge (OFC), were negative. Saline was used as the negative control for the skin prick test, resulting in only needle marks. All OFCs for nuts and buckwheat, except peanuts, yielded negative results. Given these results, it was difficult to conclude that chronic amylase elevation was indeed related to the persistence of multiple or severe food allergies other than peanuts. Amylase isozyme values from the pancreas were not elevated when the patient resumed nut consumption after the OFC (Fig. 1).

Two pathological mechanisms underlying food allergy-induced acute pancreatitis are possible. One mechanism may involve the direct infiltration of immune cells (eg, eosinophils) into the pancreas. Another possible mechanism is that edematous swelling at the ampulla of Vater, associated with mast cell infiltration, may cause occlusion of the pancreatic duct, leading to the accumulation of pancreatic juice and resulting in acute pancreatitis. This mechanism could explain the rapid decline in amylase levels reported in this patient [4, 5]. I agree with Dr. Özdemir’s comment that it is challenging to attribute the clinical picture in this case solely to the direct infiltration of the pancreas by immune cells such as eosinophils. In this patient, mild pancreatitis may have occurred through a mechanism similar to that of pancreatitis caused by common bile duct stones. In contrast to common bile duct stones, IgE-mediated swelling of the ampulla of Vater is transient and resolves quickly, leading to mild abdominal pain and rapid improvement in pancreatic enzyme levels. However, no endoscopic examinations were conducted; therefore, the definitive pathology of acute pancreatitis caused by peanut ingestion in this patient remains unknown.

In food allergies, elevated amylase levels due to pancreatic damage from ingestion are not uncommon [6]. Future research will require the recruitment and analysis of a larger number of cases, in addition to endoscopic examinations, to elucidate the definitive pathology and develop follow-up guidelines for food-induced acute pancreatitis.

## Conflicts of interest

The authors have no financial conflicts of interest.

## Author contributions

HK wrote and reviewed the letter.
